# Fibrinogen concentrate administration attributes to significant reductions of blood loss and transfusion requirements in thoracic aneurysm repair

**DOI:** 10.1186/1749-8090-9-90

**Published:** 2014-05-19

**Authors:** Koji Yamamoto, Akihiko Usui, Junki Takamatsu

**Affiliations:** 1 Department of Transfusion Medicine, Nagoya University Hospital, 65 Tsurumai, Showa, Nagoya 466-8560, Japan; 2 Department of Thoracic Surgery, Nagoya University Hospital, Nagoya, Japan; 3 Aichi Red Cross Blood Center, Seto, Aichi, Japan

**Keywords:** Massive hemorrhage, Thoracic aortic aneurysm, Cardiopulmonary bypass, Dilutional coagulopathy, Hypofibrinogenemia

## Abstract

**Background:**

Repair of thoracic aortic aneurysm (TAA) is often associated with massive hemorrhage aggravated by dilutional coagulopathy with severe hypofibrinogenemia. Although only fresh frozen plasma (FFP) is available for acquired hypofibrinogenemia in Japan, the hemostatic effect of FFP has not been enough for dilutional coagulopathy in TAA surgery. There are increasing reports suggesting that fibrinogen concentrate may be effective in controlling perioperative bleeding and reducing transfusion requirements.

**Methods:**

We retrospectively analyzed the hemostatic effect of fibrinogen concentrate compared with FFP in total 49 cases of elective TAA surgery. In 25 patients, fibrinogen concentrate was administered when the fibrinogen level was below 150 mg/dL at the cardiopulmonary bypass (CPB) termination. The recovery of fibrinogen level, blood loss, and transfused units during surgery were compared between cases of this agent and FFP (n = 24).

**Results:**

We observed rapid increases in plasma fibrinogen level and subsequent improvement in hemostasis by administration of fibrinogen concentrate after CPB termination. The average volume of total blood loss decreased by 64% and the average number of transfused units was reduced by 58% in cases of fibrinogen concentrate given, in comparison with cases of only FFP transfused for fibrinogen supplementation.

**Conclusions:**

In patients showing severe hypofibrinogenemia during TAA surgery, timely administration of fibrinogen concentrate just after removal from CPB is effective for hemostasis, and therefore in reducing blood loss and transfused volumes.

## Background

Aortic repair surgery for patients with thoracic aortic aneurysm (TAA) using cardiopulmonary bypass (CPB) is frequently complicated by massive hemorrhage, most commonly aggravated by dilutional coagulopathy with severe hypofibrinogenaemia. Patients with aortic aneurysms often show silent disseminated intravascular coagulation preoperatively [1]. Impairment of coagulation may be caused by CPB and be further aggravated by hypothermic circulatory arrest [2]. For example, the baseline plasma fibrinogen levels have been reported to decrease by 34% to 58% during CPB [3,4]. In general, failure to manage the coagulopathy and to control microvascular bleeding in cardiac surgery could lead to the increased risk of subsequent morbidity and mortality [5].

Severe hypofibrinogenemia in dilutional coagulopathy during cardiothoracic surgery causes uncontrollable oozing at multiple sites in the surgical field. This bleeding could be stopped only by quick and enough supply of coagulation factors, especially fibrinogen. As the final substrate of coagulation and the ligand of the platelet GPIIb/IIIa receptors, fibrinogen plays a key role in clot formation. Because fibrinogen is the first to fall below a critical value during massive bleeding and hemodilution [6], it would be the critical protein to be supplied first among coagulation factors. Although there are increasing reports describing the limitation of fresh frozen plasma (FFP) effect against ongoing severe hypofibrinogenemia in cardiac surgery [7,8], only FFP is currently available for acquired hypofibrinogenemia in Japan. Indeed, cryoprecipitate is not generally supplied from Japanese Red Cross and a purified fibrinogen concentrate derived from pooled human plasma (Fibrinogen HT; Japan Blood Products Organization, Tokyo, Japan) is available only for congenital fibrinogen deficiency in Japan. Fibrinogen concentrate shows a critical effect on fibrinogen recovery and subsequent hemostasis in both hereditary [9] and acquired [10,11] hypofibrinogenemic states, especially in obstetric hemorrhage [12,13], in trauma-induced coagulopathy [14,15], and in cardiovascular surgery [16,17].

The aim of this study is to examine the efficacy of fibrinogen concentrate for reduction in blood loss and transfused volume in TAA surgery. We measured the plasma level of fibrinogen at several time points in patients with TAA surgery and found that severe hypofibrinogenemia progressed during CPB. Because we hypothesized that the quick recovery from severe hypofibrinogenemia would be the most critical for hemostasis in TAA surgery, the hemostatic effect of fibrinogen concentrate was evaluated in comparison with FFP.

## Methods

In this single-center retrospective study, we analyzed the plasma fibrinogen level, total amounts of blood loss, and total transfused units of allogenic blood products in 49 patients undergoing elective surgery of thoracic aortic repair involving CPB. Any type of aortic repair surgery with root/ascending aorta, aortic arch, and descending aorta was eligible in this analysis. We administered fibrinogen concentrate when the fibrinogen level in plasma was below 150 mg/dL at removal from CPB in 25 patients. The aim of fibrinogen concentrate administration was to maintain fibrinogen levels above 200 mg/dL. The initial fibrinogen dose was 3 to 5 gram (i.e., 50–60 mg of fibrinogen/kg), but additional fibrinogen concentrate was administered repeatedly when the first administration of fibrinogen concentrate could not elevate the fibrinogen level over 200 mg/dL or achieve complete hemostasis. Meanwhile, age-matched 24 cases in the past 2 years were enrolled as the FFP group, in which only FFP was transfused for correction of hypofibrinogenemia (e.g., < 150 mg/dL) or to stop oozing after CPB termination. Red blood cells (RBCs) were administered according to institutional guidelines, e.g., for hemoglobin levels below 8.0 g/dL in active hemorrhage. Five-unit FFP was administered when the prothrombin time (PT) INR was larger than 2.0, or the patient was actively bleeding. Ten to fifteen units of PC was administered when the platelet count was below 50 × 10^3^/μl. One unit of RBC contains 130 ml of red blood cells derived from 200 ml of whole blood. Five units of FFP contain 400 ml of whole plasma, while 10 units of platelet concentrate (PC) contain 2–3 × 10^11^ of platelets, both of which were obtained by apheresis. The off-label fibrinogen concentrate substitution therapy was approved by the Institutional Review Board of Nagoya University Hospital and complies with the Declaration of Helsinki. The average volume of total blood loss and the average number of transfusion units were compared between the FFP group and the group of fibrinogen concentrate. The significant difference was evaluated by unpaired *t*-test. Pre- and intra-operative levels of hemoglobin and hemostatic markers (e.g., platelet count, PT, activated partial thromboplastin time (APTT), and plasma fibrinogen level) in each group are shown in Table 1.

**Table 1:** Parameters before TAA surgery (baseline) in each group analysed (mean ± SD)

	**Only FFP (n = 24)**	**Fibrinogen concentrate (n = 25)**
**Hb (g/dL)**	13.7 ± 2.7	14.2 ± 2.9
**Platelet (× 1000/μL)**	178 ± 57	153 ± 38
**PT (sec.)**	11.2 ± 1.3	11.4 ± 1.0
**APTT (sec.)**	33.7 ± 2.6	34.1 ± 2.8
**Fibrinogen (mg/dL)**		
**Baseline**	275 ± 66	268 ± 57
**End of CPB**	116 ± 33	108 ± 39
**End of operation**	141 ± 36	252 ± 46*

The fibrinogen levels at the end of CPB and of surgery in each group are also indicated.

*TAA*: thoracic aortic aneurysm; *CPB*: cardiopulmonary bypass; *Hb*: hemoglobin; *PT*: prothrombin time; *APTT*: activated partial thromboplastin time; *P < 0.02.

## Results

**Figure 1:**
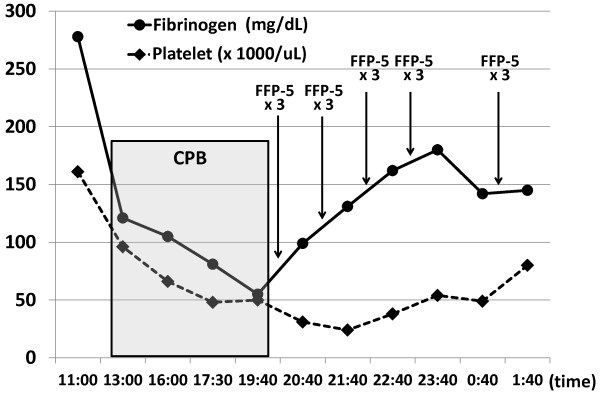
**Time course of the fibrinogen level and transfusion with fresh frozen plasma in a patient receiving TAA surgery.** The plasma fibrinogen level (mg/dL) and the platelet count are shown in a case of 71-year-old woman with dissecting aneurysm at aortic arch. The time points of administration of fresh frozen plasma (FFP) after cardiopulmonary bypass (CPB) are also indicated. The duration of CPB is shown as a gray box. FFP-5, 5 units of FFP.

The hemoglobin level and some hemostatic markers before surgery in patients showed no significant differences between the FFP group and the group of fibrinogen concentrates (Table 1). All patients analyzed in this study progressed hypofibrinogenemia during CPB and their fibrinogen levels frequently fell below 150 mg/dL at the end of CPB. Platelet counts during CPB showed lower than 50 × 10^3^/μl in most cases we analyzed, in which platelet transfusion was required. We show the representative case as Figure 1, in which only FFP was transfused for supplementation of fibrinogen. The fibrinogen level was gradually elevated by three times of transfusion with 5 units of FFP. In spite of large volume of FFP transfusion, we observed little improvement of hemostasis and continuing oozing at the surgical field, resulting in the additional massive transfusion with FFP, RBC, and PC in this case.

**Figure 2:**
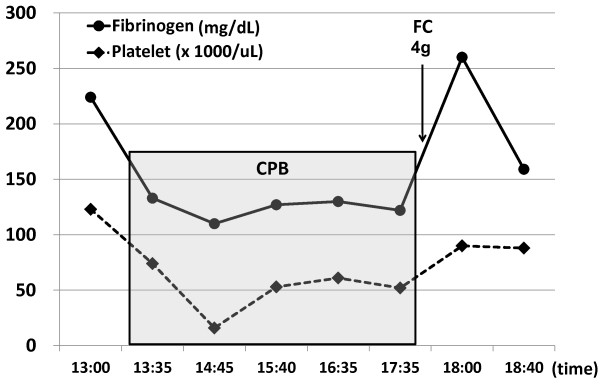
**Time course of the fibrinogen level and administration with fibrinogen concentrate in a patient receiving TAA surgery.** The plasma fibrinogen level (mg/dL) and the platelet count are shown in a case of 63-year-old man with replacement of ascending thoracic aorta. The time points of administration of fibrinogen concentrate (FC) after cardiopulmonary bypass (CPB) are also indicated. The duration of CPB is shown as a gray box.

**Figure 3:**
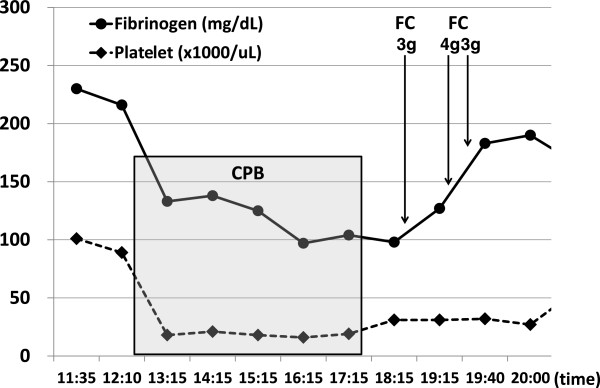
**Time course of the fibrinogen level and administration with fibrinogen concentrate in a patient receiving TAA surgery.** The plasma fibrinogen level (mg/dL) and the platelet count are shown in a case of 45-year-old man with replacement of aortic root. The time points of administration of fibrinogen concentrate (FC) after cardiopulmonary bypass (CPB) are also indicated. The duration of CPB is shown as a gray box.

**Figure 4:**
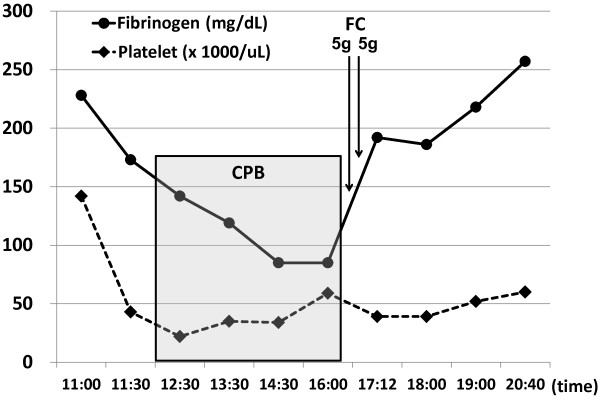
**Time course of the fibrinogen level and administration with fibrinogen concentrate in a patient receiving TAA surgery.** The plasma fibrinogen level (mg/dL) and the platelet count are shown in a case of 59-year-old man with dissecting aneurysm of descending thoracic aorta. The time points of administration of fibrinogen concentrate (FC) after cardiopulmonary bypass (CPB) are also indicated. The duration of CPB is shown as a gray box.

On the other hand, we demonstrate representative cases in which fibrinogen concentrate was administered at the end of CPB as Figures 2, 3 and 4. In the case of Figure 2, a single administration with 4 gram of fibrinogen concentrate elevated the plasma fibrinogen level by 130 mg/dL, leading to complete hemostasis in a short time. Even though severe hypofibrinogenemia around 100 mg/dL progressed during CPB in the case of Figure 3, a rapid increase in the plasma fibrinogen level and subsequent hemostasis were achieved by enough supplementation with fibrinogen concentrate. Although the fibrinogen level did not reach over 200 mg/dL in this case, no more fibrinogen concentrate was necessary because complete hemostasis was achieved after the third administration of fibrinogen concentrate. Also in the case of Figure 4, a dramatic elevation of the fibrinogen level and subsequent complete hemostasis was observed after administration with 5 gram of fibrinogen concentrate two times although critical hypofibrinogenemia below 100 mg/dL progressed during CPB. The median dose of fibrinogen administered in the group of fibrinogen concentrate was 8.2 ± 4.8 gram. In general, the fibrinogen level at the end of surgery was greater than 200 to 250 mg/dL in the group of fibrinogen concentrate administered, which was significantly higher in comparison with the FFP group (Table 1). There was no observed safety concern with using fibrinogen concentrate during and after TAA surgery.

**Figure 5:**
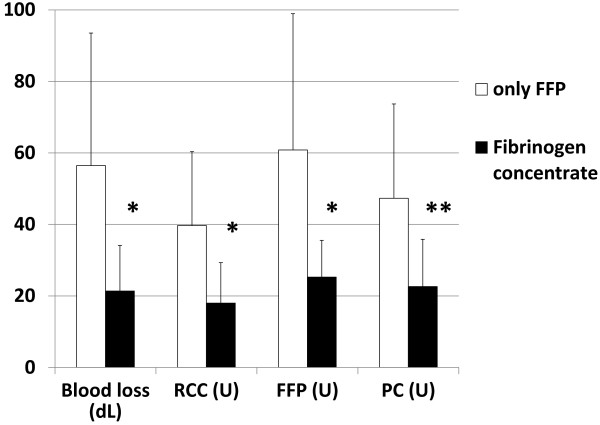
**Volume of blood loss and number of transfusion units during TAA surgery.** Open bars: cases treated with only FFP (n = 24); Closed bars: cases treated with fibrinogen concentrate as well as conventional transfusion (n = 25). RBC, red blood cell; FFP, fresh frozen plasma; PC, platelet concentrate. One unit (U) of RBC contains 130 ml of red blood cells derived from 200 ml of whole blood. Five units of FFP contain 400 ml of whole plasma, while 10 units of PC contain 2–3 × 10^11^ of platelets. The data are presented as the mean ± SD. *p < 0.05; **p < 0.01 (by unpaired *t*-test).

We compared total blood loss and transfusion volume in TAA surgery between the FFP group and the group of fibrinogen concentrates (Figure 5). Dramatic decreases in the bleeding volume and transfusion units of allogenic blood components were observed in the group of fibrinogen concentrate. In cases of fibrinogen concentrate given, the average volume of intraoperative blood loss decreased by 64% (5,640 ml in the FFP group vs. 2,140 ml in the group of fibrinogen concentrate) and the average number of transfusion units was reduced by 56% in RBC (39.7 units in the FFP group vs. 18.0 units in the group of fibrinogen concentrate), 61% in FFP (60.8 units vs. 25.3 units), and 55% in PC (47.3 units vs. 22.6 units), in comparison with cases in which only FFP was administered. Thus, significant reductions in total blood loss and transfused volume during surgery were achieved by timely administration of fibrinogen concentrate after CPB in repair of thoracic aortic aneurysms.

## Discussion

We have frequently experienced massive hemorrhage over 5,000 ml, which is characterized by microvascular bleeding due to coagulopathy [18], in surgery of thoracic aortic repair. Cardiovascular surgery using CPB decreases the plasma concentration of coagulation factors primarily by hemodilution with CPB priming and intravenous fluids [4]. We chased the plasma fibrinogen level in patients with TAA surgery and found that the fibrinogen concentration frequently fell below 150 mg/dL during CPB (Figures 1, 2, 3 and 4). In several cases, the plasma fibrinogen level reached less than 100 mg/dL at the end of CPB, as representatively shown in Figure 1 and Figure 4. The CPB-associated reduction of fibrinogen depends, in part, upon the consumptive coagulopathy deteriorated by CPB through activation of coagulation pathway primarily caused by retransfusion of blood aspirated from the surgical field [19,20]. Fibrin clots made under low plasma fibrinogen levels may be feasible and easily lyzed by fibrinolytic system, which frequently activated by CPB [21]. Thus, severe hypofibrinogenemia at the removal from CPB leads to uncontrollable microvascular bleeding, e.g., oozing at multiple sites in the surgical field, after completion of surgical hemostasis, resulting in massive hemorrhage.

Accumulating new data including this study suggest that fibrinogen plays a critical role in achieving and maintaining hemostasis, particularly in patients suffering from severe hypofibrinogenaemia during massive bleeding [22]. Because fibrinogen seems to be the coagulation factor first reaching a critically low level (100 mg/dL) even before thrombocytopenia develops during massive hemorrhage, the hemostatic therapy in this setting should be focused upon quick and enough supplementation of fibrinogen. Although, the target plasma concentration for fibrinogen replacement was predicted by *in vitro* study to be higher than 200 mg/dL as only these concentrations optimized the rate of clot formation [23], high plasma fibrinogen levels over 300 mg/dL may even compensate for low platelet counts [24]. We have conventionally used FFP for the purpose of fibrinogen replacement in intraoperative massive hemorrhage because neither cryoprecipitate nor fibrinogen concentrate has been available for decades in Japan. However, FFP has a low and variable concentration of fibrinogen and cannot be used when targeting a high plasma fibrinogen level. Furthermore, the hemostatic efficacy of FFP has been questioned [8,25]. In fact, the fibrinogen concentration was elevated by only less than 50 mg/dL after 15 units (i.e., 25 ml/kg) of FFP transfusion in TAA surgery (Figure 1), suggesting that it is difficult to reach the fibrinogen concentration over 200 mg/dL by FFP without volume overload.

Several studies and systematic reviews have suggested that fibrinogen concentrate therapy may be effective in controlling perioperative bleeding and in reducing transfusion requirements as well as blood loss in cardiovascular surgery [25-27]. The remarkable observation in our retrospective study is that administration of fibrinogen concentrate after the CPB termination sufficiently elevated the plasma fibrinogen concentration for hemostasis in TAA surgery, while only FFP transfusion did not (Figures 1, 2, 3 and 4). The plasma fibrinogen concentration reached 200 to 250 mg/dL at the end of TAA surgery in patients who received fibrinogen concentrate (Table 1). Also, it appears that enough and repeated supplementation with fibrinogen concentrate in addition to conventional transfusion therapy was strongly associated with decreased blood loss and reduced requirements of RBC, FFP, and PC (Figure 5). The difference in the volume of total blood loss between two groups may be largely attributed to the blood loss after CPB because most of blood leaking to surgical field is sucked and re-circulated during CPB. Although four products of fibrinogen concentrate, i.e., Haemocomplettan (CSL Behring, Marburg, Germany), Fibrinogene T1 and Clottagen (LFB, Les Ulis, France), Fibrinogen HT, and FibroRaas (Shangai RAAS, Shangai, China), are currently available in the world, the most widely used agent is Haemocomplettan P, commercialized as Ria-STAP in the U.S.A.. A randomized, placebo-controlled trial to investigate the efficacy and safety of Haemocomplettan P in managing severe perioperative bleeding in aortic repair surgery reported that the transfusion of allogenic blood products was significantly reduced in the fibrinogen concentrate group [28,29]. The use of Fibrinogen HT, available in Japan, in the clinical study may limit the possibility of comparison with similar trials. In any case, timely administration of fibrinogen concentrate at the fibrinogen level below 150 mg/dL after CPB termination may be the indispensable hemostatic therapy in aortic repair surgery, even if fibrinogen concentrate is not yet a standard component of many transfusion protocols. If confirmed in larger prospective randomized studies, fibrinogen concentrate would provide a concrete means of reducing transfusions and contribute to better prognosis of patients receiving thoracic aortic repair.

## Conclusions

The results of this retrospective analysis strongly support that timely administration of fibrinogen concentrate at the fibrinogen level below 150 mg/dL after CPB termination is effective for hemostasis, and therefore contributes to reduction of blood loss and transfused volumes in patients with TAA surgery.

## Abbreviations

TAA: Thoracic aortic aneurysm; CPB: Cardiopulmonary bypass; FFP: Fresh frozen plasma; RBC: Red blood cell; PC: Platelet concentrate; PT: Prothrombin time; APTT: Activated partial thromboplastin time.

## Competing interests

The authors declare that they have no competing interests.

## Authors’ contributions

KY substantially contributed to research design, analyzed data, and drafted the manuscript. AU conceived of studies and shared the overall responsibility with KY. JT revised the manuscript critically. All authors approved the submitted and final version of the manuscript.
